# First Report of the Commissioners for Inquiring into the State of Large Towns and Populous Districts

**Published:** 1844-10

**Authors:** 


### Art. XVII.

1. *First Report of the Commissioners for inquiring into the State of large Towns and populous Districts.* 1844. Folio, pp. 682.
2. *Facts and Observations on the Sanitory State of Glasgow during the last year; with Statistical Tables of the late Epidemic, showing the connexion existing between Poverty, Disease, and Crime.* By Robert Perry, m.d., President of the Faculty of Physicians and Surgeons, &c.—*Glasgow*, 1844. 8vo, pp. 38.
3. *The Glasgow Bills of Mortality for* 1841 *and* 1842, *drawn up by appointment and under the authority of the Lord Provost, Magistrates, and Town Council.* By Alexander Watt, ll.d. City Statist, &c. 1844. 8vo, pp. 106.
4. *Observations on the Epidemic Fever of* 1843 *in Scotland, and its connexion with the destitute condition of the Poor.* By William Pulteney Alison, m.d. &c. &c.—*Edinburgh and London*, 1844. 8vo, pp. 80.

I. In May of last year a royal commission was issued for an inquiry into the state of large towns and populous districts. The principal points embraced in the commission were, "the causes of disease among the inhabitants; the best means of promoting and securing the public health, under the operation of the laws and regulations now in force, and the usages at present prevailing with regard to—the drainage of lands; the erection, drainage, and ventilation of buildings; and the supply of water in such towns and districts, whether for purposes of health, or for the better protection of property from fire; and how far the public health and the condition of the poorer classes of the people of this realm, and the salubrity and safety of their dwellings may be promoted by the amendment of such laws, regulations, and usages."

It will be seen that her Majesty has, in fact, committed to the Duke of Buccleuch and his colleagues the important duty of devising a system of public hygiene for the United Kingdom. May we comment on that simple word, *important?* What should not a system of public hygiene be for Great Britain?—the land of mines and machinery; of railways and steam-ships; the land of an overflowing, industrious, energetic population; the land of strong intellect in unceasing action;—the sun and centre of modern civilization;—the beacon set up as a guide to all nations? What should not a system of public hygiene be, to harmonize with so wondrous a development of social life, and to equal so high a destiny? The duty of the commissioners is indeed important; and *that* we know they feel it to be.

This, their first Report, is confined to a statement of their proceedings, and a notice of such portions of the evidence, reports, and documentary information which best display the course and progress of the inquiry. Among the principal witnesses on the general subject are physicians. The commissioners observe:

"We would refer, in the first instance, to the evidence of Dr. N. Arnott and Dr. Southwood Smith, who have stated to us the results of their continued and latest observations; and also to the evidence of Dr. Guy, Dr. Aldis, Dr. Rigby, and Mr. Toynbee, who have had extensive practice in hospitals and dispensaries. This evidence, with that of Mr. Ward, displays their opinion and experience, that defective drainage, neglect of house and street cleansing, ventilation, and imperfect supplies of water, contribute to produce atmospheric impurities, which affect the general health and physical condition of the population, generating acute, chronic, and ultimately organic disease, especially scrofulous affections and consumption, in addition to the fevers and other forms of disease to which public attention has hitherto been chiefly directed by previous sanitary inquiries, and which are more distinctly noticed in the returns annually laid before Parliament under the provisions of the registration Act." (p. vii.)

The drainage of the metropolis was inquired into, in the first place; and a series of questions, embracing the subjects of inquiry, were also transmitted "to the municipal and other public officers in fifty towns where the rate of mortality appeared, by the returns of the registers of deaths, with a few exceptions, to be the highest. These include the largest manufacturing towns and the principal ports after London, and contain a population of more than three millions of persons. Each of these towns was afterwards visited by one of the commissioners, who examined on the spot the general condition of the town, and of the most crowded and the most unhealthy districts, making personal inquiries of the inhabitants, and hearing such statements as were made by them, or respecting them, by medical or other officers." In addition to these investigations, special inquiries were "promoted by others into the sanitary state of several towns and populous districts, more especially of those places where the growth of the population has been attended by a high rate of mortality. Some of these renewed inquiries have been of a closer and of a more comprehensive nature than those previously made, and have been conducted by persons of special qualifications, from long attention to the subject, and acquaintance with the habits and condition of the population, thus possessing the best means of insuring approximation to accuracy." A series of reports were thus obtained: on Liverpool, by Dr. Duncan; on Preston, by the Rev. J. Clay; on Nottingham, by Mr. Hawksley, civil engineer; and on York, by Dr. Laycock. In the latter, to use the words of the commissioners, "a further advance made in the investigation of the causes of mortality, is displayed in the report tracing back, for upwards of two centuries, the operation of like physical causes, in the production of different forms of epidemic disease prevalent under similar conditions, always in the greatest intensity in the same quarters in the ancient metropolitan city and county town of York."

The inquiries of the commissioners as to drainage, commence with an examination of the existing laws and of the local acts. The usages prevailing and the bye-laws in force were found, with one or two metropolitan exceptions, to be framed with a view to the maintenance of the drainage of surface water only, and without reference to house drainage and sewerage, and the constant removal of all decomposing vegetable or animal refuse. In some of the larger and more crowded towns, all entrance into the sewers by house-drains, or drains from water-closets or cesspools, is prohibited under a penalty! In scarcely one of the fifty towns examined by the commissioners can the drainage be said to be complete and good; in forty-two it is decidedly bad. The commissioners found that municipal patriots are apt to overrate the purity and excellence of the drainage in their respective towns, and to pronounce that good, which the commissioners found was only a limited drainage of a few principal streets; the more crowded portions having, in fact, no drains whatever.

An important point specially noted by the commissioners is this—that main-drains or sewers may be tolerably well formed, and subordinate or house-drains attached, but from the want of proper supplies of water, both house-drains and main-sewer act only as extended cess-pools. Of course, the miasm from many miles of extended cess-pools must be highly injurious to the health. The commissioners, therefore, next inquired into the mode in which water was supplied to towns, and also into its purity and quantity. "Upon the examination of the statements and answers from the towns to which our inquiries have been directed, it appears that only in six instances could the arrangements and the supplies be deemed in any comprehensive sense good; while in thirteen they appear to be indifferent; and in thirty-one so deficient as to be pronounced bad, and, so far as yet examined, frequently inferior in purity." Preston and Nottingham are the towns best supplied with water and at the cheapest rate.

Finding matters so bad as respects drainage and cleansing, and the necessity for amendment so obvious, the commissioners next inquired into the practicability of carrying out sanitary improvements. On the part of science and practical skill nothing seems wanting; the great obstacle is the cost of the necessary arrangements. We will allow the commissioners to speak for themselves on this point.

"We have inquired carefully as to the practicability of reducing the expenses of works for house and main-drainage, and for carrying supplies of pure water into all houses, so as to bring them within the pecuniary means of the poorest class of inhabitants. Mr. Anderton, manager of the Preston water-works, shows that the cost of new supplies may be reduced to one sixth of the former expense, if the use of water-butts be dispensed with in new districts, by the adoption of the principle of a constant instead of the present intermittent supply, and if the tenants' communication-pipes be comprehended in one contract for construction and maintenance. Mr. Quick, engineer of the Southwark Water Company, states in his evidence founded on data from experience in the metropolis, that the expense for the immediate outlay might be reduced to one fourth of the existing charge. The evidence of Mr. Hawksley exhibits the nature of the data for his important conclusion, that the result, accomplished in the town of Nottingham, is of possible attainment in many other extensive town districts in this country, and that an abundant supply of pure water may be carried into each of the lowest class of tenements, at a charge (giving a fair remuneration for the capital invested) which might not exceed 5*s*. a year, or about one penny weekly for each tenement. The same witness states, the small additional cost at which the water may be filtered, when requisite, and describes the precautions necessary to insure its purity." (p. xii.)

The same witnesses state, with reference to house-drainage, that a saving may be effected of from one half to one third of the existing charges by the substitution of impermeable tube tile drains of a superior construction, for the common brick drains, which allow the decomposing liquid refuse to permeate through the foundations.

In the Holborn and Finsbury division, " by the adoption of a system of cleansing by flushing or flooding with water, the accumulation of deposits of decomposing substances has been prevented in a large proportion of the sewers; and by rendering unnecessary the mode of cleansing by hard labour and cartage, (at once unhealthy and expensive,) 50 per cent. of the former expense has been saved."

Some interesting instances of the application of the refuse of towns thus obtained to agricultural purposes is given. The instance of Edinburgh is already familiar to the public; a similar application has been in long and successful practice at Milan, and less extensively at Ashburton in Devonshire. Mr. James Dean, who has considered the means of applying all or part of the sewerage of London to agricultural purposes, states that land worth from 30*s*. to 40*s*. per acre, when so improved, is worth from £8 to £12 per acre. At Ashburton, they have grass for the ewes and lambs a full month earlier than in other lands not so manured, at a time, of course, when lambs are the most valuable. We have reason to believe that steps are being taken to collect and apply the underground wealth of the metropolis, now lost in the Thames, to agricultural purposes. Mr. Dean's plan is to carry the sewerage of parts of London, north and east of Whitechapel road, Leadenhall street, &c. into the marshes north of West Ham and Plaistow, and thence to the river Thames below Woolwich. There he would form a series of filtering ponds, say twelve in number, of six acres and a half each. The fluid portions would be let out so as to irrigate the marshes below, of which there are several thousand acres, and the solid " barrowed" from the bottom of the settling ponds into barges. Captain Vetch devised a plan of this kind for Leeds, and estimated the *clear* annual gain at 9000*l*. sterling. Mr. Roe strongly recommended the adoption of this method to the authorities of Derby. " My opinion is," he says, " that if the sewer water were properly applied there, it would pay the expenses of the sanitary improvement of the town."

The minutes of evidence follow the Report, and occupy 470 folio pages; the appendix contains the special reports before alluded to, and other documents. We shall consider the facts they contain as contributions to hygiene, and place them as such before our readers.

*Water*. The evidence of Dr. Clark, professor of chemistry in Marischal College, Aberdeen, is first detailed. Dr. Clark distinguishes the whole saline contents of water as the neutral and the alkaline. The latter consist entirely of bicarbonates—those of lime and magnesia, and of potash and soda; the former of neutral salts of earths and alkalies. The earthy salts are, of course, the cause of hardness, but Dr. Clark has had occasion to observe that carbonic acid, in excess, renders water hard. Exposure to the air softens it, and boiling too; but this does not affect the water that is hard from neutral earthy salts. The hardness and softness have important pecuniary relations. The wear and tear of linen is much greater when washed in hard water, and, of course, the cost of soap is greater. Dr. Clark has invented and patented a method of testing the hardness of water in ten minutes. He adopts a graduated scale, in which the hardness, caused by one grain of chalk to a gallon, is expressed by one degree. He examined some of the London water; the result of his inquiry was that the hardness differed according to the state of the weather. In May 1841, the water of the Thames at Mortlake was 14·4°; of the New River 13·2°; of the East London Company's water 16·1°. In August the proportion of earthy salts was less, the degrees being 11·8°, 12·0°, and 14·1° respectively. Dr. Clark distinguishes between neutrally and alkalinely hard waters. Thus, the East London water being 16·1° of hardness, is of alkalinity 15·6°, that is, it requires as much acid to neutralize its alkalinity as 15·6 grains of chalk per gallon would require. The Thames and New River stand generally at 12° hardness and 11° alkalinity; while that of Manchester is 12° hardness and 7° alkalinity. The degrees of alkalinity express the degrees in which the water is softened by boiling. The well waters of the metropolis are much harder than that of the Thames. Torrington-square well stood at 80° of hardness; that at the gate of University college at 32°; the water from Red Lion-square pump indicates 61·5°. The artesian wells are much softer. Those at Apothecaries' Hall, at Combe's, and at Truman and Co.'s (breweries), were each about 5·5°. These last are decidedly alkaline, even to the taste when boiled: they are, in fact, solutions of carbonates of soda or potash. Dr. Clark describes his process of purifying water from bicarbonate of lime by the addition of lime water. He estimates the whole amount that might be thus precipitated from the water supplied to the metropolis by the water companies at 9000 tons per annum. The water thus purified falls to 4·2° of hardness, and acquires the same softness as if one part of the original water were mixed with three of distilled water. One hundred imperial gallons of the purified and unpurified water would require the following proportions of soap to form a lather:

| | Unpurified. | Purified. |
|---|---|---|
| East London . . . | 31 oz. | 10 oz. |
| Thames . . . . . | 28 — | 10 — |
| New River . . . . | 26 — | 9 — |
| Gade, near Watford . . . | 36 — | 9 — |

Dr. Clark estimates the annual value of soap consumed per head in London at 6*s*. 8*d*. The total value, including carbonate of soda, is £630,000, so that the difference of cost, between the supply of hard and soft water, in the matter of soap, may be estimated for London at £200,000 per annum.

*Organic remains in water.* Dr. Clark states (no doubt correctly), that the organic matter which passes into the water from the sewers is not separable by filtration. He is of opinion that undue weight has been given to its presence, and that it is assuming far too much to say that such animal matter as passes into the river remains there unchanged, especially when it is remembered how freely a river is exposed to the action of the air, and that there are other substances present, as the alkalies, so well adapted in such circumstances to increase chemical action. Professor Brande frequently found the *well* water of London contaminated by organic matter and ammoniacal salts; and this appears to be the case in other towns, as in York, for example, in the neighbourhood of churchyards. Dr. Clark found animalculæ to abound in the water of all the London companies. His attention was accidentally directed to them, and he counted, with the naked eye, no fewer than fifteen monoculi in a quarter of a pint. The impure water supplied to London is indirectly the cause of drunkenness, by exciting disgust for the pure element, and a taste for fermented drinks.

*Filtration of water.* A great variety of information under this head is accumulated in the report. Dr. Clark describes two principal modes, the one being performed by the natural filter, the other by the Lancashire filter. The first was applied on an extensive scale at Glasgow; a few miles above which city, on the bank of the Clyde, there is a very extensive convex round bank of sand used for the purpose. The Lancashire filter is the common one, with sand and gravel. Mr. Thom describes the self-cleaning filters, erected by him at Greenock, Paisley, and Ayr, which differ very considerably from the Lancashire. Over the puddled bottom of the filter a hollow floor, made of perforated tiles, is laid. Upon these are placed six successive layers of gravel, each an inch in thickness, and increasing in fineness upwards, the last being, in fact, coarse sand. Over this there is laid very fine sand to the depth of two feet, about six or eight inches at the top, being mixed with animal charcoal roughly powdered. Coloured drawings and sections are given in detail. Mr. Thom, having discovered that moss water was rendered pure and colourless by flowing over or through a particular species of lava or trap-rock (amygdaloid), was induced to substitute this rock for the animal charcoal, which he did with complete success. In an essay inserted in the appendix, Mr. Sloper argues strongly against the utility of animal charcoal, while he recommends Maurras's system.

*Supply of water to towns.* From the varied evidence of civil engineers, there appears no reason to doubt the easy practicability of supplying every house with an abundance of water at a cheap rate. In the first place, water may be kept night and day, in the pipes, in every house, at high pressure, so that no cistern would be required either for domestic purposes or water-closets; nor would there be any need for a fire-engine. At Greenock, Paisley, Ayr, Nottingham, Preston, New York, and Philadelphia, the mains being always full and at high pressure, nothing more is requisite for the extinction of a fire, than to put the hose of a fire-engine on one of the fire-plugs, which are attached to the pipes at short distances along all the streets. The water is then projected at once over the highest house. In New York, the fire insurance is 25 per cent. lower, and in Philadelphia the risk is reduced in the proportion of one to three, since the old system was abandoned. In Preston, the owners of mills are introducing water into every story, a plug and hose being placed in each. The watchman can manage the hose quite well, and the mill-owners are now so confident of the success of the plan, that they do not insure the building. In Philadelphia, the servants wash the streets and fronts of the houses by hose attached to the plugs. There can be no doubt that with a proper system of hydraulics, the whole of the refuse from the houses and streets might be washed away at very little comparative cost, or in fact with a profit, if the system of irrigation with the sewage be adopted. The desolating effects resulting from a deficient supply of water for the purpose of cleansing, as depicted by Dr. Arnott, Dr. S. Smith, and others, are strongly in contrast with the statements of Mr. Ashton, a manufacturer of Hyde, who having about 320 houses there, supplied every dwelling with water. The morals and domestic comforts of the people immediately improved.

*The atmosphere.* The vitiation of the atmosphere is manifestly one of the most important subjects of inquiry in the Report.

*Effects of deficient ventilation.* On this point we have first to notice the evidence of Dr. Arnott. Dr. Arnott reiterated the opinion he has formerly given, and gives statistical data in proof that one great cause of fever is deficient ventilation. The greater number of cases occur among workpeople, at a time when they have plenty of work (and of course plenty of food, so that starvation cannot be a cause of fever,) but at a time, too, when they are confined in their workshops, and breathe impure air for many hours daily. Evidence to this effect is quoted from the statements of the medical officer of the Spitalfields Union. At Glasgow also, Dr. Davidson observed that by far the greater proportion of cases of fever admitted into the infirmary occurred in persons in ordinary condition, to use a jockey term; indeed, only 65 were spare and 10 emaciated of 429 individuals attacked. The mortality from epidemics in Manchester and Liverpool, as in Spitalfields, diminished, not as work and consequently the means of procuring food increased, but in times of distress and as the means diminished, or, in other words, in proportion as the people, being out of work, were not confined to their badly-ventilated workshops. Of course, "distress in itself cannot be a cause of good health, but some of the consequences, such as absence from the crowded and ill-ventilated workrooms, or from ill-ventilated and ill-drained houses, and the inability to gratify hurtful and costly propensities, may, for a time, be more influential in preventing disease, than the scanty supply of food and clothing is in inducing it."

*Deficient ventilation increases the general mortality.* This subject is treated very effectively and in extenso, by Dr. Duncan, in his sanitary report on Liverpool. The mortality from fever increases with the density of the population, or, in other words, with deficient ventilation. We subjoin the following table, made up from Dr. Duncan's data, in proof. There is one portion of Liverpool in which Dr. Duncan estimates the density of the population at 657,963 persons to the square mile, nearly $2\frac{3}{4}$ greater than the maximum density of London, as stated by Mr. Farr:

| Towns. | Ratio of density of population. | Inhabitants to one death annually. | | Per centage, proportion of fever deaths to others. |
|---|---|---|---|---|
| | | From consumption. | From fever. | |
| Birmingham . . . | 40 | 207 | 917 | 4·10 |
| Leeds (Parliamentary Borough) | 87 | 209 | 849 | 4·48 |
| Metropolis . . . | 50 | 246 | 690 | 4·83 |
| Manchester (three years) . | 100 | 172 | 498 | 5·61 |
| Liverpool and West Derby . | — | — | 488 | 6·23 |
| Liverpool (Parish) . . | 138 | 156 | 407 | 6·78 |

*Influence of occupation on the health with reference to ventilation.* The evidence of Dr. Guy is principally as to the injurious effects of employments, with special reference to deficient ventilation. Dr. Guy has personally inspected the workshops of the metropolis, and carefully entered the sex, age, and occupation of several thousands of adults, presenting themselves as out-patients of King's College hospital, throwing the occupations into classes, and contrasting the results with the mortuary registers for 1838-9. The result of his researches, as they respect employments, are the following:

"1st. Consumption is relatively more frequent in persons working indoors than in those employed out of doors. 2d. In those employed within doors, it is most frequent in men using little exertion. 3d. It makes its attack earlier where it is of most frequent occurrence. 4th. It is very common in the intemperate, and in those exposed to the inhalation of dust. 5th. It is more frequent in men than in women, at least in the metropolis." (Minutes of Evidence, p 343.)

Dr. Guy states that "1st. The proportion of consumptive cases in the three classes of gentlemen (including professional men), tradesmen, and artisans (including all classes of labouring men), are respectively 1 to 5, 1 to 2·60, and 1 to 2·29; or about 16, 28, and 30 respectively in the hundred. 2d. The average age of death from consumption is earlier in the last two classes than in the first; but the artisan, who dies of consumption, dies at a somewhat later age than the tradesman." So that the tradesman of the metropolis are nearly twice as liable to consumption as the gentry, and the greater mortality, Dr. Guy thinks, is mainly attributable to their confinement during so many hours of every day in ill-ventilated shops. The slightly less liability of the artisan class is attributed to the fact that many of the latter follow employments requiring active exertion out of doors. The deaths from consumption of persons under thirty years of age, were tradesmen, 33 per cent.; labourers, in-door, 37½ per cent., out-door, 25 per cent. An indoor sedentary employment predisposes much more to consumption than all in-door active employment. Of the former, the deaths from this disease were 44 per cent., of the latter only 31½ per cent., less than the trade-class. As a special illustration of this general fact, Dr. Guy mentions the case of pressmen and compositors, "of whom the former use strong exertion in their employment, while the latter make many small movements of the hands and arms. Their rooms are generally warmed and lighted in the same way, and they both have but little space to work in, though the pressman requires more room. The comparison is greatly to the disadvantage of the compositor, who is extremely liable to consumption, and at a comparatively early period of life. This is the more striking, as the pressman is notoriously more given to habits of intoxication than the compositor."

We doubt the validity of this conclusion. The compositor follows the employment probably because being originally of delicate constitution, "light-work" was thought to be more suited to him. The compositor, too, requires more knowledge of letters (we do not mean to pun), and it is often in delicate organizations that a precocious taste for literature is developed. The bias given by congenital qualifications, whether of mind or body, to the adoption of a trade or profession, is generally forgotten: but we believe it will be found to have an important influence in determining the comparative mortality of professions. We suspect the (asserted) higher mortality of the medical profession may be explained in this way; for we can conceive nothing more conducive to health than the varied avocations of the medical practitioner.

*Scrofulous diseases result from deficient ventilation.* Mr. Toynbee gives some very important evidence on this point:

"The defective ventilation appears to me to be the principal cause of the scrofulous affections, which abound to an enormous extent among our patients. When I have had a scrofulous patient come before me, I have always been able to trace this as one of the agents. I am not prepared to state that other causes may not produce the disease, but I am prepared to state that I believe this is the greatest cause in our district. We find, as accessories, the want of personal cleanliness, badly-chosen and badly-cooked food, and defective clothing. My observation is very generally corroborative, however, of the views taken by Mons. Baudelocque, who, in a treatise, 'Etudes de la Maladie Scrofuleuse,' states that the repeated respiration of the same atmosphere is the cause of scrofula; that if there be entirely pure air, there may be bad food, bad clothing, and want of personal cleanliness; but that scrofulous disease cannot exist. He gives such facts as the following." (Minutes of Evidence, p. 333.)

Mr. Toynbee then quotes facts from Baudelocque, "who owes the details to M. Andrieux," who owes a fact, by the way, to "M. Regnault," and who quotes what Professor Alibert "has well observed." Mr.Toynbee also refers to Dr. Duncan, as adopting similar views, and as quoting "the authority of her Majesty's physician." We cordially concur in the opinion that Sir James Clark has written the best monograph on consumption in our language; but we venture to affirm that he has seen too many examples of its hereditary transmission and fatal termination in individuals who, from the moment of their birth, have had every care taken to supply them with pure air, sufficient but plain food, suitable exercise, &c., to adopt the extreme opinion of either Mr. Toynbee or M. Baudelocque. Sir James, it is true, attributes much to the want of pure air, and most correctly, but he attributes just so much and even so correctly (as Dr. Guy's statistics show), to the want of active exercise. We are not quite convinced that all the details of the school at Norwood and the Bluecoat school at Manchester are rightly stated. It is well known to practitioners connected with charity-schools, that many of their inmates are tainted with scrofula when they enter; they are the orphans of sickly parents. Mr. Toynbee's facts are, however, worthy attention, as containing much truth, and exhibit talent and praiseworthy industry. We quote a portion of his own summary, as presenting some new points. After attributing almost all infantile diseases to atmospheric impurity from defective ventilation, he says:

"Amongst other forms of disease which I think ascribable to the influence of vitiated air, is a large amount of what has not hitherto been ascribed to it, namely, deafness. In justification of this opinion, I may state that I have already made between 500 and 600 dissections of ears, with a view of determining the seat of this particular disease. 120 of these cases I have submitted to the consideration of the profession in the Twenty-sixth volume of the 'Medico-Chirurgical Transactions.' The general effect observable, is the thickening of the membrane of the middle ear. This membrane is semi-transparent, and being extremely sensitive and delicate, is, I believe, injuriously affected by the contact of vitiated air, and debilitated by it; inflammation and other diseases are induced by the access and pressure of cold air on leaving heated rooms to go out into the colder atmosphere. The delicate membrane of the ear, it is to be recollected, is longer exposed to the depressing influence of the vitiated air than any part of the body. On leaving a room the surface of the body is relieved from the continued access of the vitiated air, whilst the quantity of vitiated air contained in the middle ear remains for a considerable time, and is only slowly removed. The suspicion which I had formed from the dissections, that the cause of deafness is dependent upon the contact of foul air, appears to me to be corroborated by the fact, that at least double the number of children of the labouring classes are affected by earach and deafness than children of the rich and better conditioned classes less exposed to the like influences."

*Means of improving the ventilation of dwellings.* Mr. Toynbee and Dr. Guy have introduced plates of perforated zinc into the windows of the poor, and into some of the reading-rooms of the printing offices with the best effect. Mr. Toynbee has also introduced a cheap chimney-ventilator into rooms, contrived by Dr. Arnott. This is a square iron tube, from four to six inches in length, and three to six in diameter; one end opens into the chimney, the other into the room, and is covered with perforated zinc. Mr. Hosking objects to openings in the top of rooms; but Dr. Arnott, a great authority on these subjects, states that it is impossible to ventilate a room properly without such an opening. He has a plan in contemplation for adapting an air-pump or bellows to ventilation, not on the erroneous principle, however, of "wire-drawing" the air through small openings for its exit and entrance. Dr. Arnott thinks it would be possible to move a mechanical ventilating apparatus, at almost as little cost as to move a large clock, by winding it up, and so, for instance, supply the pure air required for a crowded evening party. A water company might supply the power by filling a cistern at the top of the houses, or a man might turn a wheel. Dr. Arnott states, (and his opinion is corroborated by Dr. Willis,) that there is much ignorance among all classes respecting the necessity and means of obtaining proper ventilation. Popular instruction is wanted. The poor stop up every crevice in winter by which cold air can be admitted. "My old teacher, Dr. Gregory of Edinburgh," Dr. Willis testifies, "in visiting the poor, used often to begin his prescription by breaking a pane or two of the window with his walking-stick, which he would make good again at the end of the illness." Men in workshops (e. g. tailors) will also stop up the ventilators. Of course, pure warm air should be thrown into the room. Few architects understand the true principles of ventilation.

*Injurious effects of an atmosphere rendered impure by miasmata.* The most obvious result of bad sewerage and drainage, by rendering the air impure, is to increase mortality and shorten life. Thus, in Leicester, in the culverted streets, the mean age of the dying was 25½ years; in those partly culverted, it was 21 years; in those unculverted, 17 years. At Chorlton-upon-Medlock, the deaths in the best-drained streets amounted to 2 per cent., or just one half of the mortality of the worst conditioned streets. In the unsewered districts in Nottingham, the annual mortality is 3¼ per cent. That much of this excessive mortality is owing to miasmata is shown by the following statement of Mr. Holland of Chorlton-upon-Medlock:

"In the third-class houses, the rate of mortality has been 2·7 per cent. in the first, and 2·8 per cent in the second-class of streets, and 4 per cent. in the worst class of streets and houses. Part of this excess must (as has been before remarked) be attributed to other causes than the bad condition of the streets; and it is difficult to determine how much ought to be attributed to this latter, the most easily removable of the causes of disease. With the view of in some degree answering such inquiry, I have ascertained what has been the mortality in twenty streets which were a few years ago in a very bad condition, and which have since been improved, and I find that in some few the rate has been higher after improvement than before, but in most of them it has been lower. The streets in question are inhabited by about 3500 persons, among whom the rate of mortality was about 3·1 per cent., or 1 in 32, before the streets were paved, and 2·53, or 1 in 39, since the improvement. There seems to be no reason for supposing that the rank of the inhabitants has altered or their number diminished. It would appear, then, that the deaths are diminished more than 20 a year out of 110, by putting the streets into proper condition." (Appendix, p. 63.)

*A miasmatic atmosphere increases the mortality from febrile diseases.* In all instances where there is an excessive general mortality and bad drainage, there is also an excessive mortality from endemic and epidemic fevers. In the culverted districts of Lancaster, for example, the deaths from this class of diseases during the year 1840 amounted to 25 per cent. of the total deaths; but in the partly culverted districts, the proportion was 33 per cent., and in the districts altogether unsewered, 50 per cent. In his report on York, Dr. Laycock adopts a juste milieu opinion; the impure air consequent on deficient drainage and ventilation acts on the lungs, predisposing them to disease, and on the system generally, predisposing it to epidemics.

"There can be no doubt that deficient ventilation, even in well-drained districts, will increase the mortality from epidemical and pulmonary diseases; but it is not generally remembered that the effects of deficient drainage are not to be avoided by good ventilation. In proof of this, the following facts will probably be deemed conclusive. The village of Rufforth, near York, is very badly drained, a wide stagnant ditch passing through the village. It is situate in a slight hollow, on a level plain, bounded by Marston moor, Askham bogs, &c. The village of Acomb is about two miles distant from Rufforth, and is situated on an eminence overlooking the level. Both of course are well ventilated, as country villages usually are; but Rufforth being less populous and more agricultural, has of the two the advantage in this respect.

| | Population in 1841. | Altitude in feet. | Inhabitants living to one annual death, from | | Mean age at death. |
|---|---|---|---|---|---|
| | | | All causes. | Epidemics. | |
| Rufforth . . | 276 | 61 | 34 | 69 | 28 |
| Acomb . . | 774 | 110 | 41 | 258 | 35½ |

"The scarlatina, when epidemic at Rufforth, was so malignant as to be fatal in a few hours, and was termed by the villagers the 'black' fever."

The general summary of York exhibits similar results:

| | Mean Altitude. | Population to square Rood. | Mean age at Death. | Inhabitants to one death annually. | | | Deaths of Labouring Class per cent. |
|---|---|---|---|---|---|---|---|
| | | | | From all causes. | From Epidemics. | From Pulmonary Disease. | |
| Best drained and ventilated Parishes | 50 | 27 | 35·32 | 54·32 | 347·72 | 334·22 | 40·2 |
| Intermediate Parishes | 43 | 40 | 27·79 | 41·41 | 247·20 | 219·70 | 52·5 |
| Worst drained and ventilated Parishes | 33 | 63 | 22·57 | 32·15 | 129·48 | 153·00 | 62·8 |

In addition to these modern instances, Dr. Laycock has appealed to historical data, and has drawn up a report on the epidemics of York, especially those prevalent in the sixteenth, seventeenth, and eighteenth centuries. "From this it is manifest," to quote Dr. Laycock's conclusions, "that the frightful mortality of these plagues and 'visitations,' as they were termed, was altogether dependent on the malaria generated in the city, partly from absolute uncleanliness, partly from deficient sewerage and drainage. It also shows that the seasons of prevalence, and the localities of previous epidemics (the plague in 1551, the plague of 1604, and the cholera of 1832,) are, to a great extent, identical with those in which they still flourish in a mitigated form, although the insalubrious localities now principally occupied by the poor were then the residences of the wealthier classes of society." Under what hygienic conditions does the fever of our large towns prove most fatal? It will have been observed that deficient ventilation was considered to be the principal cause of fever by Dr. Arnott; we shall now adduce some facts to show that the more usual and efficient cause is an impure atmosphere in consequence of deficient drainage, either alone, or combined with deficient ventilation. Dr. Southwood Smith, physician to the London Fever hospital, was asked if his statement was correct, that fever constantly prevails, generated by the dirt and filth in which the people habitually live? "That is too true," was the pithy answer.

"In every district in which fever returns frequently, and prevails extensively, there is uniformly bad sewerage, a bad supply of water, a bad supply of scavengers, and a constant accumulation of filth; and I have observed this to be so uniformly and generally the case, that I have been accustomed to express the fact in this way. If you trace down the fever districts on a map, and then compare that map with the map of the commissioners of sewers, you will find that, wherever the commissioners of sewers have not been, there fever is prevalent, and on the contrary, wherever they have been, there fever is comparatively absent." (Minutes of Evidence, p. 69.)

The miasm is blown with the wind in a certain direction, and fever breaks out to leeward, and travels by jumps like the cholera.

"Every now and then, we meet with a positive and precise fact, which shows that fever prevails in a neighbourhood just as the wind blows from an infected place in the direction of that neighbourhood, and that the production of any new cases of fever entirely ceases as soon as the wind shifts. Many most instructive observations of this kind are on record, and I have given one or two examples of them in the report alluded to; but a large and crowded town does not afford favorable opportunities for such observations. What we more commonly observe in the metropolis is, that when once fever breaks out in one of the districts we have been speaking of, it continues to rage there during the prevalence of the epidemic; it does not spread in any very clear and remarkable manner to an adjoining district, but runs over it, and breaks out in a distant oue.

"As in cases of cholera? Very much so." (Minutes of Evidence, p. 76.)

Although Dr. Duncan (in his report on Liverpool,) is inclined to doubt whether the malaria arising from decomposing animal and vegetable matter "is an efficient cause of fever, independently of other circumstances," we shall press his facts into our service, and turn his own guns upon himself. Moisture, of course, is requisite to the formation of malaria or miasm.

"The following table shows that the proportion of damp and wet cellars is greatest, and least in the same districts respectively, when fever reaches its maximum and minimum; and that the three most unhealthy districts generally are the most unfavorably situated in this respect; the proportion of damp and wet cellars in the former being 44½ per cent., and in the latter 28½ per cent. of the whole. Other diseases are probably also more prevalent in cellars, for the total number of dispensary patients residing under ground is certainly much larger than the cellar proportions of the working classes should give; but that fever especially infests these abodes, is shown by the fact (so far as a few hundred cases can be trusted to for the purpose,) that while of every 100 dispensary patients of all descriptions attended by Mr. Higginson and myself, 31·22 resided in cellars, there were 36·22 in cellars, out of every 100 cases of fever." (Appendix, p. 26.)

The table that follows shows, in fact, what Dr. Duncan asserts. Then again Dr. Duncan makes this statement, clinching it with a table: "The prevalence of fever, and the rate of mortality proceed inversely as the efficiency of the sewerage," and yet adds, "I am inclined to look upon the absence of sewerage, although certainly one element of mortality, as less influential in its action than some others which have been noticed." But why so inclined? His facts are good and true; are corroborated by other observers, and are in accordance with the general pathology of all malarious fevers. Perhaps Dr. Duncan has set up an idolon specus, an idol of the den.

*Injurious effects of a miasmatic atmosphere on the morals. Infanticide.* There are other injurious effects of a malarious atmosphere generated in towns. The most striking of these is the infantile mortality among the poorer classes. The cause appears to operate by rendering the infantile epidemics more virulent, or by inducing a general sickly state, and so increasing the general mortality from natural causes. This sickly state also increases the mortality from poisonous doses of opiates. The child is peevish by day and by night; the nurse, hired by the mother to tend her infant, while she labours at the factory, is peevish too, and wants peace; or at night, the parents are wearied and want rest. Recourse is therefore had by both nurse and mother, to "infant's quietness, one teaspoonful at the age of three months," "Godfrey's quietness, dose twelve to fourteen drops," "soothing syrup," &c. Sales of these opiate mixtures for infants by fifteen venders residing in Ashton-under-Lyne, amount to the enormous quantity of nearly seven gallons weekly!

*Drunkenness* is another of the moral effects of a malarious atmosphere in towns. In quoting the following interesting statement from Dr. Southwood Smith's evidence we put in a caveat to this effect, that the peculiar and strong desire for stimulants observed to be endemic among a poor civic population, is due in some degree to their more excitable nervous system; and that this excitability is constitutional and developed not simply by exposure to injurious agents, but also by the greater mental, and less muscular activity of the citizen.

"The poison generated in these neglected districts, and to which these poor persons are habitually exposed, is a sedative poison, among the most distinctive characters of which are the depressing effects produced by it both on body and mind. This is one of the main causes, not only of the mental apathy, of which I have already spoken, but also of that physical listlessness which makes them incapable of any great exertion. Every one who has observed his own sensations during the few days which precede an acute attack of fever, can well appreciate that feeling of *malaise*, more intolerable than pain; and it is no wonder that they fly to anything which affords a prospect of temporary relief from it.

"When you speak of stimulants, do you mean that it drives them to the use of ardent spirits? Yes, and some to opium.

"Is not opium now used to a great extent? To a much greater extent than any one could credit who is not aware of the fact." (Min. of Evidence, p. 73.)

Here our notice of the contents of this Report must end. We need scarcely say that we have done it only inadequate justice. There are several statistical important documents to which we have made no allusion whatever. One of these is a table exhibiting the standard of vitality actually attained in middle-class life. The society of Friends was adopted for this inquiry, and from the table, it appears that the mean age of the dying, during the years 1841-1843, belonging to this society was 48·80 years for males, and 55·13 years for females. We have reason to believe that Mr. Chadwick undertook the necessary inquiries. We ought to state that an octavo edition of the Report has been printed.

II. Our readers have been already made acquainted with the course and nature of the epidemic which prevailed during last year in Edinburgh, Glasgow, and other cities and towns of Scotland. The causal dependence of this fever upon poverty is the theme of Dr. Perry's pamphlet. The following proem to Dr. Perry's argument may be considered as containing its substance.

"The question has been frequently asked, what is the cause of the present epidemic? Respecting the immediate cause of any epidemic disease with which a community may be visited, very little satisfactory information has hitherto been obtained; so that to give a definite answer in our present state of knowledge is beyond our power. Attempts have indeed been made, and those who have the least experience generally speak with most certainty on the subject. At present it is the fashion to ascribe every epidemic, whether malignant fever, cholera, dysentery, or influenza, to malaria arising from decaying animal or vegetable substances, owing to the want of sewers for carrying off such substances, and the scanty supply of water. There are perhaps few places better supplied with water than Glasgow; and I have observed on more than one occasion during the prevalence of malignant fever, that its progress was equally rapid and violent during a period of intense frost, when everything has been covered with snow, and the whole liquid substances in the streets firmly bound up for weeks together, without the possibility of any putrefaction going on. This was particularly the case in 1837, when the frost continued very intense for upwards of six weeks, when the number of fever cases was greatest; the same thing was observed at Moscow during the prevalence of cholera. It appears that some epidemics are *spread solely by means of infection;* these are specific poisons, generated in the bodies of those who are undergoing the disease, and spread either by contact or by the emanations from the bodies of those affected by the specific disease. This is the case with all that class of infectious fevers called exanthemata; as small-pox, measles, scarlatina, and typhus. There are other epidemics which seem to depend on some peculiar state of the atmosphere predisposing the bodies of those whose constitutions are weak to suffer under their influence, of which the present epidemic affords a good example. This brings us to the more practical part of the question; viz., the causes which predispose persons to be affected by any prevailing epidemic; and in this inquiry very little difficulty presents itself, there are so many facts which attest that it is the poor and indigent part of the population who furnish the earliest, and by far the greatest amount of victims." (p. 6.)

As we differ in part from Dr. Perry, and as we think it of great importance that those who like our author, and (we may venture to add) ourselves, are labouring to ameliorate the sad and disgraceful condition of the labouring population of the kingdom should have union of sentiment and object, we will shortly state the points on which we differ; premising, however, that we admire and fully appreciate the ardent philanthropy and active benevolence displayed in this as well as in Dr. Alison's and Dr. Watt's pamphlet. In the first place, we think that Dr. Perry has not exactly comprehended the views of those who, like ourselves, trace an intimate connexion between malaria or miasmatic emanation and epidemical diseases. These views have been stated in the preceding pages. We consider that that peculiar state of the atmosphere alluded to by Dr. Perry, as predisposing the bodies of those whose constitutions are weak to suffer from epidemics, consists in the presence of malaria or miasmata. If it were an accurately ascertained fact, (which it is not,) that the late epidemic in Glasgow, and epidemics in general, was or are equally rapid and violent during a period of intense frost, it would not be absolutely opposed to our views, since persons who have resided in a malarious atmosphere, retain the morbid predisposition alluded to, during a period more or less prolonged after a removal to a purer air. The ague, for example, will first appear several days, in the traveller, after exposure to malaria, who has only traversed a marsh for an hour or two. We readily grant that the exanthemata spread solely by infection; but the malignancy of their type and their mortality will be in a direct ratio, cæteris paribus, to the impurity of the atmosphere breathed by those suffering from them. It is for this cause, that the value of fever hospitals is doubtful in proportion to their magnitude, although we were hardly prepared to find, as is stated by the district surgeons, that the mortality was less in the filthy hovels of Glasgow than in the fever hospital of that city. Glasgow may be absolutely better supplied with water than most other places, yet not better supplied relatively. And if ever so well supplied, of what value can the supply be, if not used? What do the district surgeons say on this point—the use of water in removing decomposing organic remains? Of district No. 4, Mr. Smith, the surgeon, remarks,

"The tenements in which I have visited are occupied from the cellars to the attics, and almost altogether kept for lodging-houses, many of them being more fit for pig-styes than dwellings for human beings; and in not a few the donkey and pigs rest at night in the same apartment with the family. The entrance to these abodes is generally through a close, not unfrequently some inches deep with water, or mud, or the fluid part of every kind of filth, carelessly thrown down, from unwillingness to go with it to the common receptacles; and in every close there is at least one of these places situated, often immediately under the windows of the dwelling-houses, or together with byres, stables, &c., forming the ground-floor; while the stench arising therefrom in summer, pollutes the neighbourhood, and more especially renders the habitations above almost intolerable." (p. 20.)

Dr. Brown, of district 11, observes,

"The whole of the Burnside, especially the ground-floors, are not fit places for pigs; height of ceiling about four feet, and at almost every flood in the Clyde, they are inundated by the Molindinnar Burn; every inhabitant of these dens has had fever." (p. 25.)

Dr. Fisher, of district 12, states,

"No. 23, a long dirty close. In one house at the top of it several severe cases occurred. Access is obtained to this house, or rather apartment, by an outside stair, by the side of which is a dunghill, the stench from which is intolerable. I have seen the dung reach the landing-place on the top of the stair. I attended for fever almost every individual residing in the front land. The number was very great." (p. 27.)

While the above extracts strikingly exhibit the coexistence of the sources of malaria and fever, the following shows that by removing the sources, the district-surgeon, Mr. Clark, checked the spread of the epidemic.

"In No. 100, I caused the policemen on the station, to summon every tenant in the close before the police magistrate. The result was a thorough cleansing out of the filth which had accumulated for months, and a diminution of the fever cases to less than one half in about a week." (p. 23.)

As decomposing vegetable and animal remains are to be so generally found *within* the abodes of the poor of Glasgow, it might be inferred *à priori*, that the fever would be scarcely less prevalent in the winter than the summer months. On comparing, however, table iii of six districts in Glasgow, in which the fever was most numerous, and in which epidemics generally are most prevalent, with table i, in which the deaths in each month from May to December are given, we find that the numbers attacked monthly, describe a curve, the highest convexity of which corresponds to the two epidemical months of August and September. The number of coffins given out by the poors' house monthly, exhibits a similar curve. The average of May and December, the two extremes being 113, of August and September, 236. From these considerations, we infer that the malarious atmosphere of the closes of Glasgow accelerated the progress and increased the mortality of this epidemic. As usual, the Irish exhibited their national predisposition to suffer from fever. In Dr. Paterson's district at least one third of the patients belonged originally to Ireland. Dr. Perry states, "in 1832 the proportion of Irish to the whole pauper population admitted into the fever hospital, was 33 per cent., in 1842 it amounted to 41 per cent."

As a remedy for the evils he so vividly depicts, Dr. Perry strenuously advocates the adoption of sanitary measures, basing his arguments partly on the mere money saving.

"As comparatively few of the sufferers belong to the middle ranks—being, as already remarked, chiefly confined to the poor and labouring classes, a large proportion of them being adults—some idea will be formed of the amount of suffering among the poor, by considering that, on an average, they were unable to follow their usual employment, or gain anything for their support for five weeks; and, calculating their weekly earnings at the small sum of four shillings weekly, it exhibits a positive loss to the poor, in eight months, of not less than £32,000. Had this been the result of a strike among the workmen, the whole newspaper press would have been filled with lamentations; but being the result of causes over which the poor had no control, the press has been comparatively silent, and little has been done for their relief. When it is considered besides, that Glasgow has not suffered alone, but that the epidemic has visited nearly every large town in this country, the effect ought to call forth some effectual means of relief, and the devising of measures for preventing the recurrence of a similar or even worse calamity, which, in the present circumstances of the country, may with certainty be predicted." (p. 12.)

Dr. Perry enters his solemn protest against the doctrine, that the medical treatment of the poor at their own houses was less expensive than in the hospital. The epidemic attacked 11½ per cent. of the population; and he asks, "if the public had possessed the means of isolating the first cases as they appeared, by sending them to a hospital, would the epidemic not have been checked? And in place of having 32,000 cases, with all their accompanying sufferings, might it not have been limited to less than 5000?"

Dr. Perry proposes the establishment of a board of health. His plan differs little from those which have already been found to be altogether inefficacious to meet the exigencies of the case. The Report of the Health of Towns' Commission will, we have no doubt, attract Dr. Perry's attention, and probably induce him to modify his views, both with respect to the causes of disease, and the legislation requisite for their removal.

Another point to which Dr. Perry adverts, as intimately connected with the poverty and destitution of the poor, is their mental and moral degradation. This intimate connexion with, or dependence of vice upon extreme poverty, is too obvious to those who have any intercourse with the poor, he justly observes, to require remark. We cordially agree with him in his opinion, that to attempt to remove the one while the other is allowed to remain in large cities, is to attempt impossibilities. He compares the dietaries of English workhouses and of the prisons with the full diet of the Glasgow Infirmary, and shows that it is barely equal to the ordinary diet of the former, and the lowest rate of diet in the latter, namely, 24 oz. of wheaten bread per day. Statements from the report of Mr. Hill, inspector of prisons, are quoted, from which it appears that at one time forty persons, the majority of them able bodied, were undergoing voluntary imprisonment. Indeed it is obvious as an axiom, that the prison must be a palace, in its domestic arrangements, when compared with the dreadful residences of the Glasgow poor.

Many of our readers will have read the first volume of the Sydenham Society, and learnt something of the terrific ravages of the "black death." If a malignant influenza, as that epidemic appears to have been, were to break out in Glasgow we are firmly of opinion that nothing would stay its ravages, except want of victims. It would sweep through that and similar towns like a hurricane, leaving behind it the silence of desolation. A lavish expenditure, extorted by national anguish, would then be useless. Boards of health would wrestle in vain with its gigantic strength. The danger must be anticipated. One thing is to us manifest: whatever evils are consequent on the English poor-law, (and that, like all human institutions, it has its evils, we readily acknowledge,) the evils of *no*-poor-law in Scotland are greater. Wherever a poor-law exists, and is efficiently worked, human nature cannot possibly sink into that abyss of degradation, in which, according to Dr. Perry's statements, the poor of Glasgow are plunged.

We cannot conclude our notice of Dr. Perry's brochure without stating, in his own words, the circumstances under which it was printed:

"In order to aid the laudable design of Dr. Hutchinson in exercising the mental and bodily faculties of the inmates of the Lunatic Asylum, the printing of this paper, the colouring of the maps, &c., is wholly the work of the inmates. It is hoped that this circumstance will induce the reader to make allowance for any thing he may observe amiss in the manner in which this, their first essay, has been performed."

Although the printing is not first-rate, it is extremely creditable to the printers. The only mishap worthy of notice is "the pie" they have made of the author's academic and professional titles. If they had had better type and paper, the book would really have been a stylish affair.

III. Now and then we meet with works so admirably condensed, as to defy even our powers for greater condensation. Dr. Watt's pamphlet is one of these. Justice could only be done to it by transferring two thirds of its contents, word for word, to our pages. We therefore recommend the vital statist to do well by placing it at once in his library; and to our general readers we will present some of the more important points connected with our present subject—the public hygiene of Great Britain.

*Effect of the malarious atmosphere of Glasgow.* We ought to premise, that Dr. Watt, like Dr. Duncan, Dr. Perry, and Dr. Alison, is of opinion, that the cause of fever is to be sought for rather in the want of nutritious food than of pure air. But Dr. Watt, although warring on the side of the philanthropic Alison, is a moderate man—a genuine eclectic, like ourselves; and insists that destitution is a principal cause of fever, rather to defeat the opponents of a poor-law for Scotland, than to defend an "idolon specus." As we have no doubt that, sooner or later, a poor-law *must* be introduced into Scotland, we shall, as in the case of Dr. Duncan, turn some of his guns against himself.

*Effects of Glasgow malaria on the spread of fever.* Dr. Watt makes an estimate (he says it is a moderate one,) that 60,000 persons in Glasgow have suffered from fever during the last seven years. According to our views, heat and moisture, and organic remains in a state suitable to decomposition, are necessary to the production of malaria, and of course to the fevers aggravated by or dependent on malaria. Heat and moisture and dirt may be *inside* a dwelling (as they are in Glasgow in many thousands of dwellings,) during the winter; but in summer, heat and moisture and filth are both outside and in. Then, consequently, *cæteris paribus*, there must necessarily be abundant cases of fever in Glasgow, and more abundantly in summer than in winter or in spring. What says Dr. Watt?

In January 1837, the deaths from fever and influenza were 10·19 per cent. of the whole deaths in Glasgow and its suburbs; but during the five months beginning June 1843, the proportion was 33·38 per cent. We have taken these two years for comparison, for the purpose of illustrating a general principle, overlooked, we observe, by Dr. Alison and his coadjutors, namely, that an epidemic fever will cease to spread, independently of all other considerations, from a want of persons liable to its attacks. Yellow fever and all the exanthemata are of this kind, not omitting specially the exanthematous typhus. Now, in 1837 the fever was at its height in the winter months, 1972 persons having died in January only; but in 1843 it was at its height in the autumnal month of October. In the former year, *relief began to be administered to the destitute just when the fever was beginning to decline*, namely, in February; in the latter year the relief *ceased to be given just when the fever began to increase*, namely, in May. Hence Dr. Watt, adopting the principle of "*post hoc, ergo propter hoc*," is warranted in inferring that fever and destitution were necessarily allied; but, according to our reasoning, the two were only coincident. The fever would have gone on, in 1843, concurrent with the relief, had that been continued.

*Effects of the malarious atmosphere of Glasgow on diarrhœa.* Cholera, dysentery, and diarrhœa are the plagues of summer camps, and of large towns in which the heats of summer act upon moist and decomposing debris. Glasgow, as we have learnt from Dr. Perry's pamphlet is full of these; and accordingly we expect to find that in the summer months (fever being absent,) there will be an excessive number of deaths from "bowel complaints" in that city. What do Dr. Watt's statistics teach us?

"But among the more important diseases which fall under our consideration, in treating of the vital statistics of Glasgow, are those which are classed under the head of bowel complaints; as the mortality by these is higher in Glasgow than it is in either Dundee, Perth, or Edinburgh, as will be seen by the following abstract; and it will be found that it is to these diseases that the high mortality among children in this city, during the months of August and September, are to be ascribed.

Proportion of Deaths by Bowel Complaints, for a series of years, to the mean population of those years.

| | In Glasgow. | In Dundee. | In Edinburgh. | In Perth. |
|---|---|---|---|---|
| Bowel complaints | 0·370 | 0·263 | 0·167 | 0·171 |

......... "The amount of deaths by bowel complaints, however, is very different in the several months. In January, the deaths by these diseases amount only to 8·57 per cent. of the whole deaths during the month, and to 0·031 per cent. of the mean population. In August, they amount to 18·40 per cent. of the whole deaths, and to 0·047 per cent. of the mean population, which accounts for the mortality under five years of age, this month amounting (table lxx) to 50·47 per cent. of the whole deaths during August, and to 0·130 per cent. of the population. In September, the deaths by bowel complaints amount to 17·53 per cent. of the whole deaths, and to 0·060 per cent. of the population." (p. 87.)

Dr. Watt remarks that "the uniformity in the high amount of mortality by bowel complaints, during the months of August and September, does not appear to be accidental, as the deaths by this disease are in excess for these months for all the years noticed in the Tables, with the exception of 1838." He hints, too, that the preeminence which Glasgow and Dundee have in the number of deaths from these forms of disease may be attributable in some degree to their preeminent filthiness. Dr. Watt must not forget, that the *natural* drainage of a town has a decided influence on the amount of malaria exhaled from the surface. Edinburgh may, therefore, be superior to the other two towns, because it has a superior *natural* drainage.

*Effect of the malarious atmosphere of Glasgow on infant life.* All the reports made to the health of towns' commission show that bad sewerage, and an excessive number of deaths among children under five years of age are coincident; there is no exception to this principle: Glasgow, of course, is no exception. During the five years ending 1841, 44·58 per cent. died, aged under five years, and 49·16 per cent. in 1842. In Liverpool, during the years 1839-41, according to Dr. Duncan's report, 52·8 per cent. die at these ages. It is in the deaths from epidemics, however, that the effects of civic malaria on infant life are best exhibited. At Glasgow, the months most fatal to children are August and September —the malarious months. Above one half of the whole deaths are children under five years.

"On an average of five years, 84·06 per cent. of the whole deaths by that disease [bowel complaints,] takes place in Glasgow under two years of age, and, as above stated, 90·69 per cent. under five years of age." (p. 88.)

Dr. Watt's statistics of the exanthematous epidemics are equally instructive. They shall first show the influence of locality:

"The following facts go far to show that it is to the circumstances of the people, and to the local condition of towns, that we are to look for a greater or less mortality by these diseases. There are many circumstances common to Glasgow and Dundee, both as regards the circumstances of the majority of the people and the local condition of the towns. There are also many circumstances in common to Edinburgh and Perth in these respects; and the mortality in them, by eruptive diseases, is not so very different, as will be seen from the totals of the following abstract, which gives the proportion of deaths per cent. by these diseases in each town for a series of years, to the mean population of these years:

| | Edinburgh. | Perth. | Glasgow. | Dundee. |
|---|---|---|---|---|
| Measles | 0·075 | 0·092 | 0·198 | 0·177 |
| Scarlet fever | 0·052 | 0·070 | 0·096 | 0·120 |
| Smallpox | 0·056 | 0·050 | 0·144 | 0·140 |
| Total Eruptive Diseases | 0·183 | 0·212 | 0·438 | 0·437" (pp.86-7.) |

Next the influence of temperature shall be shown:

"By summing the proportions, as shown in table lxxii, for the eruptive diseases, measles, scarlatina, and smallpox together, it is found that in January, on the average of these seven years, 13·96 per cent. of the whole deaths are occasioned by them. It will be farther seen, that the proportion of deaths which occur by these diseases gradually diminish till April, when the proportion of deaths by them is only 11·48 per cent. of the whole deaths during that month. It will likewise be observed, that the proportion that they bear to the mean population gradually diminishes also. From April, the proportion of deaths, though somewhat suddenly in May, gradually rises in amount each succeeding month till November, in which month they appear to be excessive, amounting to 19·33 per cent. of the whole deaths, and to 0·048 per cent. of the population. In December, the proportion of deaths by these diseases falls to 16·99 per cent., which is still considerably higher than it is in January." (p. 86.)

Some of Dr. Watt's tables give us data for estimating the influence of climate on epidemics; we have not room for these, but we can assure our readers that Glasgow and Edinburgh are no better than Philadelphia and New York.

We have read Dr. Watt's tables, and remarks thereon, with much interest. The tables of monthly deaths give a much more satisfactory account of the influence of atmospheric changes than our registrar-general's quarterly division. We regret to observe that Mr. Cartwright has adopted the quarters instead of the months in his report on the seasonal mortality of Preston. The comparison of the seasonal statistics of Preston and of Glasgow would have been interesting. Meteorological tables for 1841 and 1842 are appended to the volume.

IV. The object of Dr. Alison's pamplet, is not sufficiently described by its title; its general scope is to show the necessity and economy of a poor-law for Scotland. It is, however, to the connexion of destitution with fever that we shall limit our remarks.

We think much of the controversy respecting the causal relations of poverty (for that is what Dr. Alison means by destitution) to fever, has originated, partly, in an indeterminate use of words, partly in misconceptions. Dr. Alison has, "on different occasions, adduced evidence to prove, not that destitution is an adequate cause for the *generation* of fever, nor that it is the *sole* cause of its extension, but that it is *one* cause of the diffusion of fever; of such power, that an epidemic of that disease, invading a community where the provisions against destitution are inadequate, is very generally found to spread, *cæteris paribus*, to an extent remarkably greater than where adequate provisions of that kind exist." The truth of this proposition, we think, will be granted by all medical practitioners; want of food predisposes the system to receive the febrile contagion. Dr. Alison states, "It will be at once perceived, that the relation which I maintain to exist between destitution and fever is not simply that of cause and effect, but that of *predisposition*, favouring the effect of another cause, *which is essentially variable*. Where destitution exists, it prepares victims for fever, but the fever 'bides its time.'" And yet, in a note in the preceding page, Dr. Alison states:

"I reply that the question before us is not, what are the causes of destitution, but whether destitution is a cause of fever? Supposing all the destitution which the medical practitioners and benevolent individuals alluded to have seen, to have been the effect of misconduct, still, if they have seen fever spread with unusual rapidity in such families, they are entitled to infer, that destitution (caused by misconduct) is a cause of fever; and if so why not destitution caused by misfortune?—of the still more frequent existence of which with us, I have given, and shall give, more than sufficient evidence." (Note, p. 3.)

That certainly is the question as we have generally understood it, but do not the preceding extracts exhibit some inconsistency? Has Dr. Alison, like lesser men, an *idolon specus*? and sometimes, when facts are presented to him while doing his devoirs to his idolon, are they offered up to it? Thus, in a report by the physicians of the Infirmary it is observed that "the fever which now prevails is *unquestionably connected very closely with circumstances peculiar to the destitute part* of the population;" and from the noun-adjective "destitute," Dr. Alison infers the circumstances to consist in destitution. Yet could not Dr. Arnott say, and say justly, that deficient ventilation constituted *the* "circumstances," and triumphantly point to the identical fact, in support of his views, which Dr. Alison quotes triumphantly in support of his, namely, that the inhabitants of the highest stories offered the greatest proportion of fever victims? Or, again, would not Dr. S. Smith say that *the* circumstances were deficient cleansing and sewerage? because wherever fever prevails most, there is the least amount of sewerage to be found. It appears to us, that the predisposing causes of fever are found in all agencies which debilitate the system, or are allied to the proximate cause, the materies morbi. Several of these may, indeed in towns do, coexist, as the malarious emanations arising from stagnant sewerage, deficient nourishment, and a deficient supply of pure air. Malaria will cause fever we know; deficient ventilation will propagate it; innutrition will render it more fatal. But fever is not the only head of this hydra; need we mention scrofula and phthisis as the other two? We thus recommend to our readers Our idolon specus—the idol of Our den.

When the Queen's commission is fulfilled, and the cause of diseases among the people ascertained, what will the government do? We presume, it will take steps to enforce the supply of sufficient light, pure air, and pure water to the crowded population of our towns: A poor-law for Scotland will enforce a supply of food. From the general tenor of the inquiry already made, we presume, too, that the British system of public hygiene will be made, as far as practicable, by the conversion of the refuse of towns to agriculture, a self-supporting system,—an imitation, in miniature, of nature's own processes, which admit of no waste.

---

